# 

**Published:** 1885-09

**Authors:** 


WHOLE NUMBER, CCLXXX. SEPTEMBER, 1885. NUMBER 2. VOLUME XXV.

### THE BUFFALO Medical AND Surgical Journal

**EDITORS:**

**THOS. LOTHROP, M. D.** **A. R. DAVIDSON, M. D.**

*ASSOCIATE EDITORS:*

F. S. CREGO, M. D. C. C. FREDERICK, M. D.
F. R. CAMPBELL, M. D. H. MICKLE, M. D.

PUBLISHED BY THE MEDICAL JOURNAL ASSOCIATION.
No. 5 WEST CHIPPEWA ST.

Entered at the Post-Office at Buffalo, N. Y., as Second-class Mail Matter.

